# Electrochemical Sensor for Determination of Various Phenolic Compounds in Wine Samples Using Fe_3_O_4_ Nanoparticles Modified Carbon Paste Electrode

**DOI:** 10.3390/mi12030312

**Published:** 2021-03-17

**Authors:** Pwadubashiyi C. Pwavodi, Vasfiye H. Ozyurt, Suleyman Asir, Mehmet Ozsoz

**Affiliations:** 1 Department of Biomedical Engineering, Near East University, Near East Boulevard, 99138 Nicosia, Cyprus; mehmet.Ozsoz@neu.edu.tr; 2 Department of Food Engineering, Near East University, Near East Boulevard, 99138 Nicosia, Cyprus; hazal.ozyurt@neu.edu.tr; 3 Department of Materials Science and Nanotechnology Engineering, Near East University, Near East Boulevard, 99138 Nicosia, Cyprus; suleyman.asir@neu.edu.tr

**Keywords:** electrochemical sensor, Fe_3_O_4_ nanoparticles, carbon paste electrodes, sinapic acid, syringic acid, rutin, voltammetry, electrochemical impedance spectroscopy

## Abstract

Phenolic compounds contain classes of flavonoids and non-flavonoids, which occur naturally as secondary metabolites in plants. These compounds, when consumed in food substances, improve human health because of their antioxidant properties against oxidative damage diseases. In this study, an electrochemical sensor was developed using a carbon paste electrode (CPE) modified with Fe_3_O_4_ nanoparticles (MCPE) for the electrosensitive determination of sinapic acid, syringic acid, and rutin. The characterization techniques adapted for CPE, MCPE electrodes, and the solution interface were cyclic voltammetry (CV), differential pulse voltammetry (DPV), and electrochemical impedance spectroscopy (EIS). Scan rate and pH were the parameters subjected to optimization studies for the determination of phenolic compounds. The incorporation of Fe_3_O_4_ nanoparticles to the CPE as a sensor showed excellent sensitivity, selectivity, repeatability, reproducibility, stability, and low preparation cost. The limits of detection (LOD) obtained were 2.2 × 10^−7^ M for sinapic acid, 2.6 × 10^−7^ M for syringic acid, and 0.8 × 10^−7^ M for rutin, respectively. The fabricated electrochemical sensor was applied to determine phenolic compounds in real samples of red and white wine.

## 1. Introduction

Phenolic compounds contain a broad class of flavonoids and non-flavonoids phenols, which occur naturally as secondary metabolites throughout the plant kingdom. They spread widely into several taxonomic groups and play structural and protective functions in plants [1,2]. Flavonoids and phenolic acids contain at least one aromatic ring with one or more hydroxyl groups attached to them. They have a wide range of structures and can be classified based on the number and arrangement of carbon atoms. Flavonoids are classified into flavonols, flavones, flavan-3-ols, anthocyanidins, flavanones, isoflavones, and others, while the non-flavonoids are classified into phenolic acids, hydroxycinnamic acids, hydroxybenzoic acids, stilbenes, and other. These phenolic acids are commonly conjugated to sugars and other organic acids [3]. These phenolic compounds are used in food processing due to their properties associated with color, flavors, preservatives, and antioxidants that improve human health [4]. Phenolic compounds have a ubiquitous presence of different proportions in plant-based foods. Daily consumption of products such as fruits, wines, vegetables, grains, teas, spices, and coffees improves human health. They improve human health through their radical scavenging activities, which provide an anticancer effect against atherosclerosis, inflammatory diseases, and other oxidative damage diseases and control oxidation in the human body [4,5,6,7,8]. These phenolic compounds undergo electrochemical oxidation at modified electrodes through the following basic principles: (1) Phenols oxidize to phenoxy radicals in one-electron, one-proton irreversible step. (2) The anodic oxidation of phenolics depends on the stability of the generated phenoxy radical. (3) The formed phenoxy radicals that are unstable co-exist in three resonant forms, the ortho and para-phenoxy radicals formed, which have larger spin density and stability that undergoes secondary chemical reaction hydroxylation. (4) When an additional electroactive –OH group is added at the ortho and para positions, the process leads to the production of two-electron and two-proton in a pH-dependent reversible process, which has higher stability and appears at a less potential than that of the meta-di-phenol or mono-phenol. (5) Non-electroactive substituents that are present produce small oxidation peak potentials shift while a greater shift is observed when the substituents at the para and ortho-positions are linked. (6) Substituents that have their electrons removed have a higher anodic peak potential shift, while substituents that are electron donor make oxidation process easy. (7) The electrochemical oxidation process involves the participation of protons; the higher pH values, the easier the electron loss [4,9]. Other researchers have published works on rutin [10,11,12,13,14,15], but less or no work is reported on sinapic acid [16] and syringic acid. These phenolic acids contain oxidizable phenolic substituents on the aromatic ring or reducible olefinic bond, which is why their voltammetric determination [16,17]. Different methodologies have been used for the analysis of phenolic compounds from different origins, which includes high-performance liquid chromatography (HPLC) in reversed-phase under UV detection, liquid chromatography with mass spectrometry (LC-MS) via electrospray ionization (ESI), gas chromatography (GC) with flame ionization (FID) and MS detection, capillary zone electrophoresis (CZE) under UV direct detection, and capillary electrophoresis coupled to mass spectrometry (CE–MS) [2,5,18,19].The techniques mentioned above used for phenolic compounds determination are sensitive and selective; however, they present some disadvantages. They include a large amount of sample needed for analysis, complex procedures for sample determination, time-consuming procedures, and the pretreatment process for the sample is unfriendly. In comparison to the above traditional instrumentation, electrochemical methods have the advantage of high sensitivity, selectivity, ease of use of instruments, low cost of preparation, and simple and rapid detection of a low amount of sample [7,13,15,20].

Modified electrodes in electrochemical analysis for sensitive and selective detection of compounds have been widely used [21]. CPE has many advantages over other solid electrodes, which include their biocompatibility with test samples, a fast and straightforward method of preparation from cheap materials. They possess a reproducible and renewable surface, which presents low residual current during analysis. CPE also has low production cost, porous surfaces, and can be used for miniaturization in electrodes for electrochemical sensors [21,22]. However, MCPE with nanomaterials has recently shown a substantial increase in the electrochemical properties of the analyzed compounds. The main advantages of using MCPE with nanoparticles over macro electrodes or unmodified CPE are effective surface area, increased sensitivity and selectivity, and effective mass transport by mediating electron-transfer between electroactive species during reactions in solution [23].

Nanoparticles are in small sizes, ranging from 1–100 nm, and they possess chemically, physically, and electronically unique properties that make them different from those of bulk materials. These different properties allow them to be utilized in various analytical methods, where they are employed to fabricate novel and improved sensing devices such as in electroanalytical sensors. Nanoparticles have been widely used to modify electrodes used in sensitive and selective detection of biological compounds in analytical methods. The application of nanostructured materials to these electrodes indicated considerable improvements in the electrochemical behavior of compounds because of their high effective surface area, catalytic effect, and mass transport [24,25]. Fe_3_O_4_ nanoparticle belongs to the class of nanoparticles, and they are used for modifying electrodes because of their excellent electrochemical properties [25]. They are used to modify the working electrode to enhance detection limit, provide large electroactive surface area, catalytic effect, high electromagnetic activity, attractive electron transport, sensitivity, and chemical stability [26]. The Fe_3_O_4_ nanoparticles also offer a conductivity effect, making it suitable for enhancing the electron transfer between analytes and electrodes. Fe_3_O_4_ nanoparticles have significant application areas in biomaterials, bioseparation, biomedical and bioengineering, and food analysis [27].

**Figure 1:**
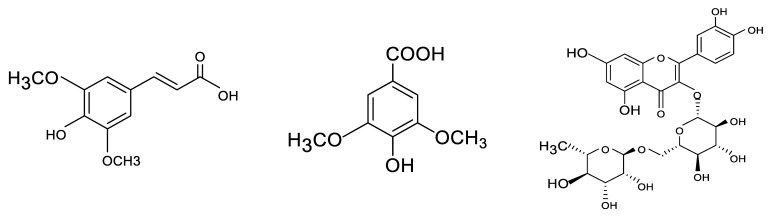
The chemical structures of sinapic acid, syringic acid, and rutin, accordingly.

This research aims to study the electrochemical behavior of various phenolic compounds (Figure 1) by fabricating an electrochemical sensor using carbon paste electrodes modified with Fe_3_O_4_ nanoparticles. This study is the first report on using Fe_3_O_4_ nanoparticles to modify carbon paste electrodes for the electrochemical determination of sinapic acid, syringic acid, and rutin based on our careful check of works reported on the detection of these phenolic compounds. CV, DPV, and EIS analyses were performed as characterization studies for CPE, MCPE, and the solution interface. Scan rate and pH studies were performed as optimization studies. A rapid validation test was carried out using gold screen-printed electrodes, and the result was compared to CPE and MCPE. The electrochemical sensor was applied in real samples of red and white wine to determine the presence of phenolic compounds.

## 2. Materials and Methods

### 2.1. Chemicals and Reagents

**Figure 2:**
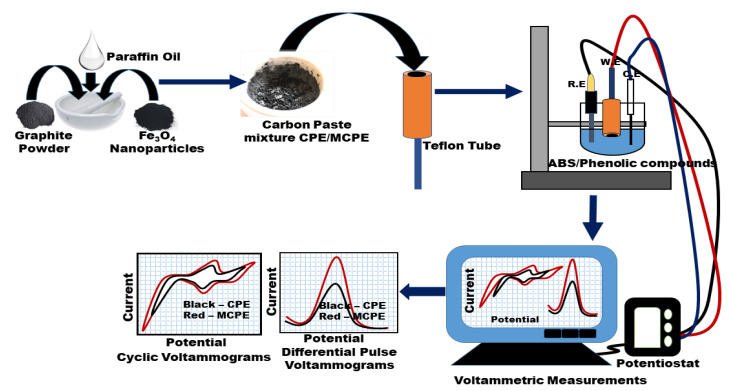
Schematic diagram of the phenolic compounds electrochemical sensor preparation.

The powdered phenolic compounds (sinapic acid, syringic acid, and rutin), paraffin oil, and carbon powder were all procured commercially from Sigma-Aldrich, Istanbul, Turkey. Fe_3_O_4_ nanoparticles powder was purchased commercially from Sigma-Aldrich, Istanbul, Turkey. They have a particle size of 50–100 nm, scanning electron microscopy (SEM) for surface characterization; Brunauer–Emmett–Teller (BET) surface area analysis is 6–8 m^2^/g, melting point of 1538 °C, titration by Na_2_S_2_O_3_, % of Fe is 71.5%, an appearance of black color, powder form and spherical shape, the density of 4.8–5.1 g/mL at 25 °C, a bulk density of 0.84 g/mL, the purity determined using trace metal analysis is 97% (≤35,000.0 ppm), a quality level of 100, Inductively coupled plasma (ICP) major analysis confirms iron component. All reagents were of analytical standards and used as obtained (Figure 2). The pH value of the acetate buffer solutions (ABS) used for the study is 0.5 M, ABS pH 4.8. The stock solutions of the phenolic compounds were prepared with ultra-pure water at a concentration of 1000 ppm (4.5 × 10^−3^ M for sinapic, 5.1 × 10^−3^ M for syringic, and 1.6 × 10^−3^ M for rutin). The stock solution was then diluted into standard concentrations of 200 ppm (0.9 × 10^−3^ M for sinapic acid, 1.0 × 10^−3^ M for syringic acid, and 0.3 × 10^−^^3^ M for rutin), which were used as working solutions. The wine samples used for the analysis were commercial brands of wine (Angola kavaklidere—dry white wine— and dikmen kavaklidere—dry red wine) purchased from a market.

### 2.2. Instrumentation and Methods

All the voltammetric measurements were performed using the potentiostat–galvanostat (AUTOLAB-PGSTAT204, Metrohm, Utrecht, The Netherlands) and operated with Nova 2.1.2 software. The potentiostat–galvanostat was connected to a three-electrode system cell, a carbon paste electrode, and Fe_3_O_4_ nanoparticles modified carbon paste electrode as working electrodes. Ag/AgCl/3 M KCl was used as a reference electrode and a platinum wire as an auxiliary electrode in a 10 mL cell containing 0.5 M ABS pH 4.8 as supporting electrolyte. The pH measurements were all carried out with an edge H12002 pH meter (Hanna Instruments, Woonsocket, RI, USA).

### 2.3. Fabrication of Bare CPE and MCPE

The ratios of carbon powder to paraffin oil (binder) were compared for best results using the ratios 70:30 and 60:40 (wt/wt%), respectively, and 60:40 ratio was taken as the optimized proportion for the study. The carbon paste mixture, as the control (CPE), was prepared by hand, mixing 60 mg of carbon powder with 40 mg of paraffin oil to obtain a homogeneous mixture of 60 vs. 40 mg. The carbon paste mixture prepared with Fe_3_O_4_ nanoparticles (MCPE) contained 60.0 mg carbon powder, 30.0 mg of paraffin oil, and 10.0 mg of Fe_3_O_4_ powder in a ratio of 60:30:10 (wt/wt/wt%) [23]. The homogenous pastes were packed to fill two different 4 mm diameter cavity of Teflon tubes, one for the CPE and the other for the MCPE. A copper wire for conductivity was connected to the end of the electrodes (Teflon tubes). The surfaces of the electrodes were polished by smoothening them with a smooth paper to obtain a smooth and crack-free surface. After each analysis, new electrode surfaces were prepared by inserting the paste into the Teflon tubes and their surfaces polished. This process was repeated (Figure 2) throughout the experiments before each new measurement.

### 2.4. Voltammetric Measurements

The voltammetric techniques used for the study were CV and DPV. The measurements of these analytes at the electrode surfaces were carried out in ABS 0.5 M, pH 4.8. The scan rate study was done using CV, by varying the applied scan rates at a range of 0.03, 0.06, 0.09, 0.12, 0.15, 0.18, 0.20, 0.25, 0.3, 0.35, and 0.40 V/s. The pH study was done using CV by varying the pH values at a range of 2.6, 3.8, 4.8, 5.6, 6.5, 7.4, 8.4, and 9.2 pH, respectively, using a scan rate of 0.2 V/s. The DPV analysis was done from 0.0 V to 1.0 V. EIS measurements were performed in the frequency range of 100 kHz–0.1 Hz, in a redox solution of 5 mM [Fe(CN)_6_]^3−/4−^ containing 1 M KNO_3_. At CPE and MCPE; the electroactive surface area of CPE and MCPE was determined in 1 mM K_4_[Fe(CN)_6_], which was used as an electrochemical redox probe in 0.1 M KCl. The same condition was used for the scan rate study by varying the scan rates from 0.1, 0.12, 0.14, 0.16, 0.18, and 0.2 V/s, employing the Randles–Sevcik equation.

### 2.5. Preparation and Detection Procedure of Real Samples (Red and White Wine)

DPV technique was used to analyze phenolic compounds’ content in spiked samples of the red and white wine samples. The voltammograms produced were recorded using a method of standard addition of serial dilutions of known volumes and concentrations of the phenolic compounds (sinapic acid, syringic acid, and rutin). A volume of 1 mL of the wine samples only was inserted into a 10 mL beaker and was completed with ABS of (0.5 M, pH 4.8) to a volume of 10 mL. Aliquots of the standard phenolic compounds from (0.03 × 10^−3^–0.05 × 10^−3^ M) were then added to the 10 mL beakers having 1 mL of the wine samples and completed to 10 mL with ABS. After which, they were stirred for two minutes with a magnetic stirrer. Measurements from the DPV analysis were recorded from each beaker that contains the wine and aliquots of the standard phenolic compounds [23].

## 3. Results and Discussion

### 3.1. Electrochemical Behavior of the Phenolic Compounds at CPE and MCPE

**Figure 3:**
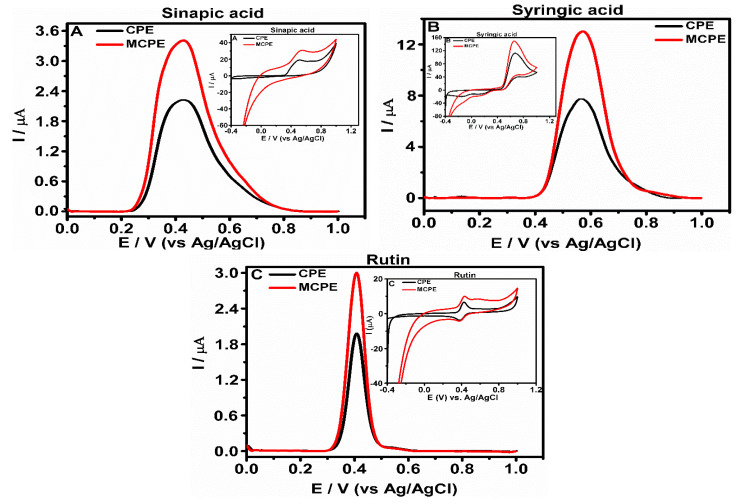
Differential pulse voltammograms (**A**–**C**) show the determination of the electrochemical behavior of the selected phenolic compounds of 0.9 × 10^−3^ M for sinapic, 1.0 × 10^−3^ M for syringic, and 0.3 × 10^−3^ M for rutin at carbon paste electrode (CPE) and carbon paste mixture prepared with Fe_3_O_4_ nanoparticles (MCPE) in 0.5 M ABS with pH 4.8, recorded at 0 V to +1.0 V. The inset cyclic voltammograms (**A**–**C**) of the selected phenolic compounds at CPE and MCPE was carried out in 0.5 M acetate buffer solutions (ABS) with pH 4.8 and recorded at a scan rate of 0.2 Vs^−1^.

The electrochemical behaviors of the selected phenolic compounds at the CPE and MCPE surface were studied using CV and DPV analysis. The differential pulse voltammograms (Figure 3) and the inset cyclic voltammograms show the electrochemical behavior of the analytes at the surface of electrodes. The phenolic compounds show visible peak currents higher in MCPE and lower in CPE [21,22,28]. These electrochemical behaviors of the analytes observed from the peak currents, visibly shifting, could be suggested to be a result of the nanoparticles at the electrode surface increasing the current signal as a result of the catalytic effect of the nanoparticles, thus making the current signals to increase in the modified electrodes more than the unmodified electrodes [23].

**Figure 4:**
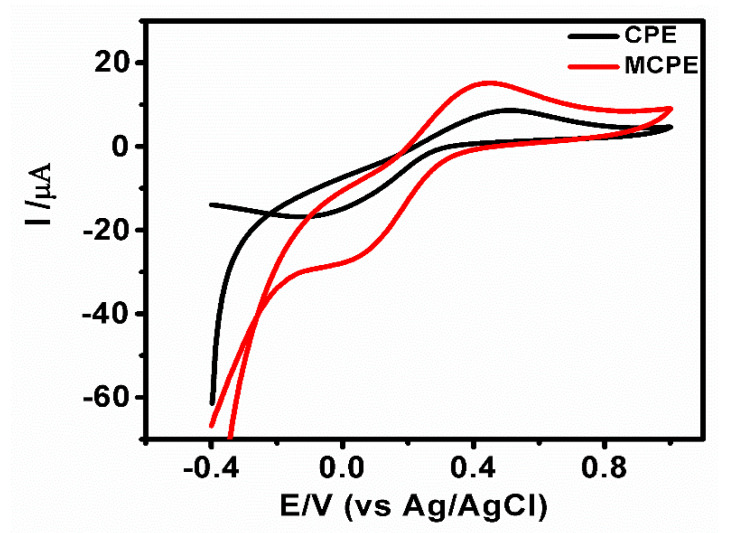
Cyclic voltammogram shows the response of 1 mM K_4_[Fe(CN)_6_] at the CPE and MCPE at a scan rate of 0.1 V/s.

The anodic peak potentials (E_pa_) and cathodic peak potentials (E_pc_) observed from the CV analyses of the phenolic compounds (inset Figure 3) showed positions of oxidation and reduction potentials of the analytes. Sinapic acid presented positions of one E_pa_, while syringic acid and rutin presented two positions of E_pa_ and E_pc_; the reduction peaks showed low current peak heights that are observed on the reverse scan. This behavior suggests that the oxidation reaction’s product undergoes a further chemical reaction for syringic acid and rutin or is not reduced at the carbon paste electrode for sinapic acid [21]. As the peak current is higher in MCPE than CPE, the shoulders of the peaks observed from MCPE are also broader than the CPE. This behavior can be suggested to be a result of increased electroactive surface area by incorporating the Fe_3_O_4_ nanoparticles [23], which is similar to the results obtained from the determination of the electroactive surface area of the electrodes (Figure 4 and Figure S2). If the modified electrode functioned as an electrocatalyst or the reaction was electro-catalyzed, there would have been a reduction in the peak potential, which suggests a reaction that is faster with a less overpotential [23]. The voltammetric behavior of the phenolic compounds is shown to agree with the chemical reaction proposed globally for phenolic group oxidation in aromatic compounds [23,24,25]. The peak heights of the anodic peak current (I_pa_) and cathodic peak currents (I_pc_) of the analytes at CPE are lower compared to that of the MCPE, suggesting that the activity which occurred at the surface of the CPE is poor and less than the MCPE [21]. The presence of Fe_3_O_4_ nanoparticles in the MCPE supports the transfer of electrons, enhances the current response, and can support the adsorption of the analyte and its enrichment onto the surface of the electrode, thereby promoting the oxidation process [29]. The peaks obtained through DPV are shown to be better defined and have high sensitivity to low concentration of analytes and lower background current when compared to the results obtained using CV.

The hydroxy groups of the phenols are oxidized through the transfer of two electrons, which form a quinone group after the liberation of 2H^+^. The phenolic compounds with one anodic peak indicate an electrochemical behavior, which suggests an oxidation reaction that leads to the formation of a stable quinone group, which is reduced on the reversed scan (Figure S1A). This is also similar to the hydroxy group’s oxidation of other phenolic compounds at their ortho position [30]. However, the phenolic compounds with two anodic peaks indicate the formation of a semiquinone radical in the first step. The second peak corresponds to the oxidation of the semiquinone to the quinone group. The Fe_3_O_4_ nanoparticles provide stability for the complete oxidation of the phenolic compounds [21,31] (Figure S1B).

### 3.2. Evaluation of the Electroactive Surface Area

The electroactive surface area of the electrodes CPE and MCPE were determined using CV in 1 mM K_4_[Fe(CN)_6_], which was used as an electrochemical redox probe in 0.1 M KCl. MCPE displayed an enhancement of the current response (Figure 4), which indicates that the CPE’s electrochemical active sites were increased on surface modification by the Fe_3_O_4_ nanoparticles. The MCPE presented a larger current response (I_pa_ = 15.20 μA) in comparison to the CPE current response (I_pa_ = 8.64 μA); this can be attributed to the electrocatalytic activity and enhancement of the modified surface area. The cyclic voltammograms of CPE (Figure S2A) and MCPE (Figure S2B) show that the oxidation and reduction potentials were shifted to more positive and more negative potentials, respectively, with a linear increase of the redox peak current as the scan rate is enhanced from 0.1 to 0.2 V/s. The plot of Ipa versus υ^1/2^ (Figure S2C,D) shows linearity with an R^2^ value of 0.9963 for CPE and 0.9830 for MCPE. The electrodes’ electroactive surface area was estimated according to the slope of Ipa versus υ^1/2^ for a known concentration of K_4_Fe(CN)_6_ using the Randles–Sevcik equation [32].

I_pa_ = 2.69 × 10^5^n^3/2^AC_o_D^1/2^υ^1/2^ (1)

I_pa_: indicates anodic peak current (A), n: the number of electrons exchanged during the redox process, which is presumed to be equal to one, A: surface area of the electrode (cm^2^), C_o_: concentration of the redox probe (mol cm^−3^), D: diffusion coefficient assumed to be equal to 6.23 × 10^–6^ cm^2^ s^–1^, and from the slopes of I_pa_-υ^1/2^ relation, the microscopic electroactive surface area was calculated to be MCPE (0.043 cm^2^) in comparison with the CPE (0.015 cm^2^). The results show that the presence of Fe_3_O_4_ nanoparticles increased the active surface area of the electrode.

### 3.3. Effect of pH on the Phenolic Compounds Oxidation at CPE and MCPE

**Figure 5:**
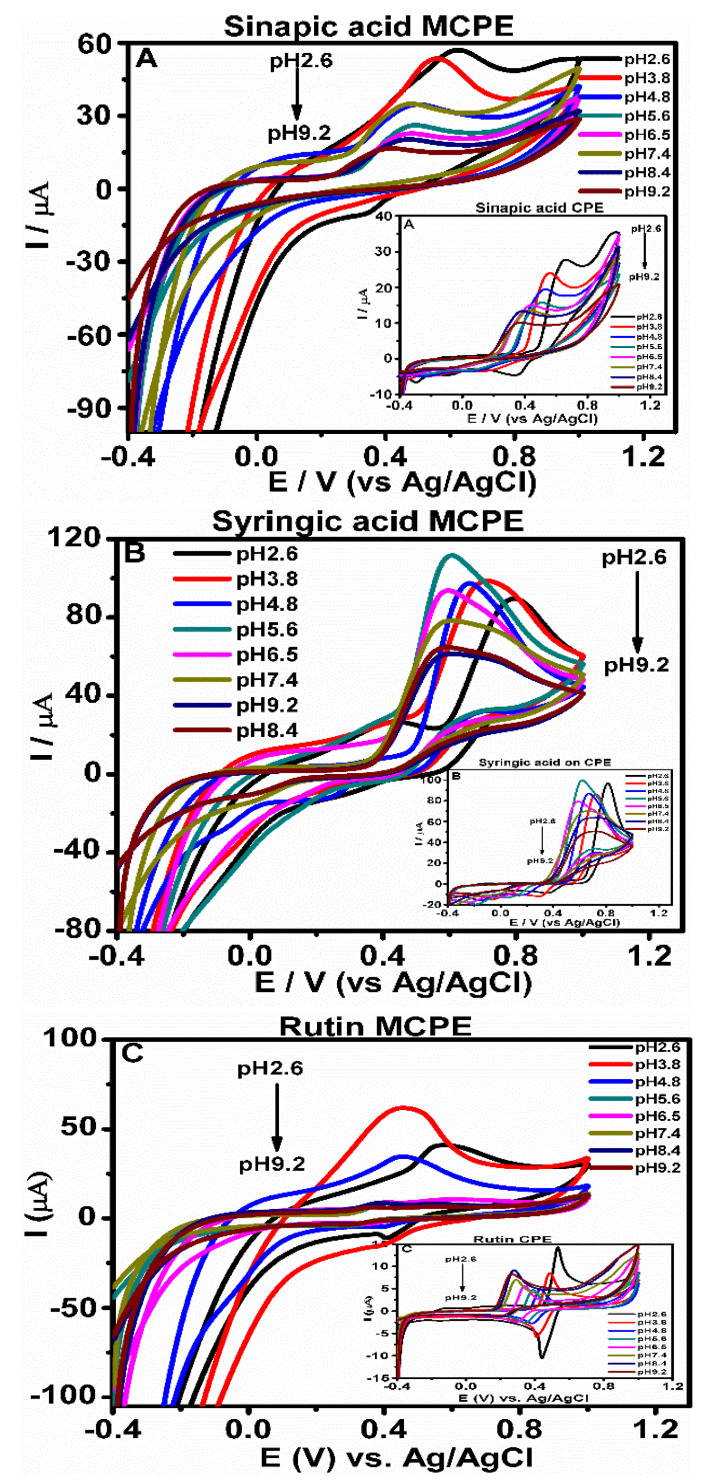
Cyclic voltammograms (**A**–**C**) show the effect of pH on the electrochemical behavior of the phenolic compounds, in 0.5 M acetate buffer of pH 2.6, 3.8, 4.8, 5.6, 6.5, 7.4, 8.4, and 9.2, at a scan rate of 0.2 V/s with a reversible scanning potential range of −0.4 to 1.0 V. The inset figures show cyclic voltammetry (CV) at CPE.

The effect of pH of the buffer solution on the current response of phenolic compounds oxidation at CPE and MCPE was studied using CV to observe their electrochemical behaviors (Figure 5). The pH of the different buffer solutions affected the oxidation activity of the phenolic compounds on the surface of CPE and MCPE, thereby causing changes to the electrochemical behavior of the phenolic compounds. This effect can be seen (Figure 5), as the anodic peak currents and potentials of the phenolic compounds on the CPE and MCPE showed a progressive decrease with increasing pH values from 2.6 to 9.2 [33]. The cyclic voltammograms of the phenolic compounds showed a clear pH dependence of their electrochemical behavior at the surface of the electrodes, as the increase in the pH of the ABS gradually lead to a decrease of the anodic peak current.

As the buffer solution’s pH increases, there is a gradual negative shift of the peak potentials, which shows a linear relationship between the pH values and E_pa_ [33]. The relationship between the anodic peak potentials E_pa_ and the pH values were studied, and the plots produced showed a linear regression relationship with an equation having values for the phenolic compounds presented in Table 1 and Figure S3. All of the values produced from the slope of E_pa_/pH of the regression line are compared to the Nernstian value of 59 mV/pH and 29.5 mV/pH, which shows the number of electron and protons involved in oxidation/reduction reaction for two-electron/two-proton process and two-electron/one-proton process [30,34,35,36] The electrochemical behavior could further be explained by the fact that at low pH value, the concentration of the analytes protonated form oxidized is high and increases with decreasing the pH [37]. This supports the ease of oxidation reaction and enhances mass transport at the surface of the electrode. When the pH is increased, the current begins to gradually decrease, which could be suggested to be a result of the decrease of the protonated form concentration [37].

### 3.4. Effect of Scan Rate on the Phenolic Compounds Oxidation at CPE and MCPE

**Figure 6:**
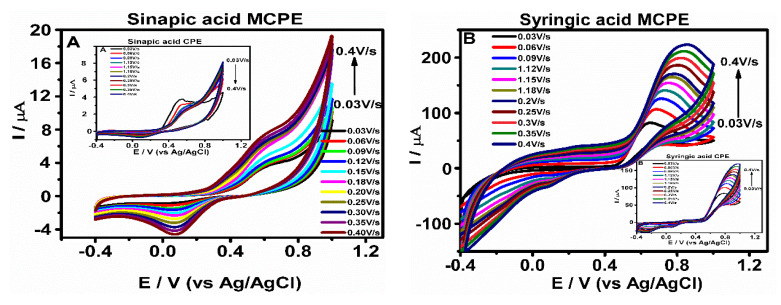

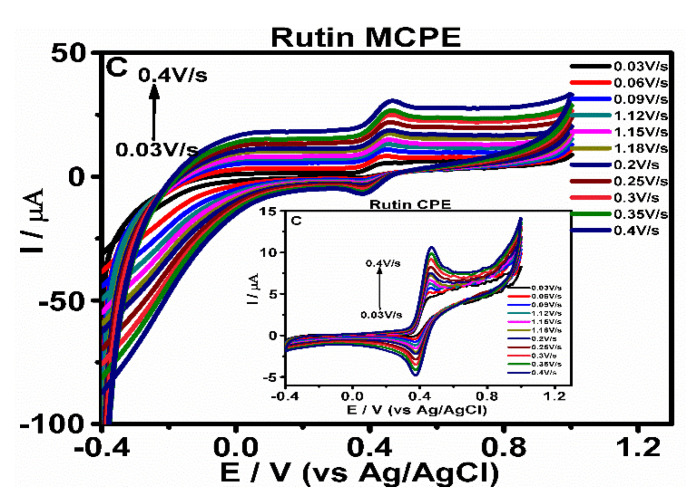
Cyclic voltammograms (**A**–**C**) show the electrochemical oxidation and reduction behavior of phenolic compounds at CPE and MCPE in 0.5 M of ABS having pH 4.8 and scan rates of 0.03, 0.06, 0.09, 0.12, 0.15, 0.18, 0.20, 0.25, 0.3, 0.35 and 0.40 V/s, respectively, with a reversible scanning potential range of −0.4 to 1.0 V.

The influence of scan rate on the electro-oxidation behavior of the phenolic compounds at CPE and MCPE surface was demonstrated using CV (Figure 6). The voltammograms show an increase in the peak current signals with increased applied scan rates. The values measured for the peak current were used for plotting linear equation of peak current Ip versus square root of scan rate ν^1/2^, which indicated a typical diffusion-controlled reaction (Table 2 and Figure S4). Another plot of the peak currents Ip versus scan rate (ν) both for anodic and cathodic peak currents using same experimental conditions was performed and yielded a straight line (Table 2 and Figure S5) which is typical for adsorption controlled. As the scan rate applied increases, the peak currents for anodic and cathodic also increase linearly, indicating a quasi-reversible oxidation reaction [38].

The logarithm of anodic peak current and logarithm of scan rate (log I_pa_ versus log ν) approach was used to confirm whether the electrochemical reaction at the electrode surface is diffusion or adsorption controlled (Table 3 and Figure S6) using linear relationship plots. The values of the slopes obtained were close to 0.5, which is attributed to electrochemical reactions that are diffusion-controlled [39,40,41].

### 3.5. Characterization of CPE and MCPE Using EIS

**Figure 7:**
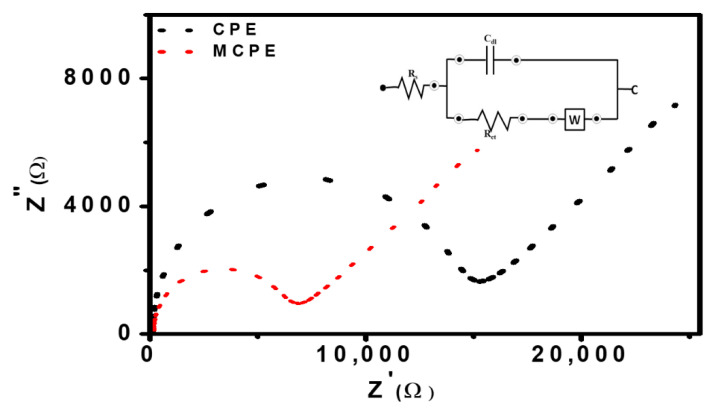
Nyquist plots represent the EIS measurement performed in the frequency range of 100 kHz–0.1 Hz, in a redox solution of 5 mM [Fe(CN)_6_]^3−/4−^ containing 1 M KNO_3_ using CPE and MCPE for surface characterization. The inset figure is the equivalent circuit showing resistors and capacitor (c).

The EIS was used to study the difference in the behavior of the CPE and the MCPE. This method is an effective tool used to study the electrode/solution interface properties and how charge transfer occurs between the redox solution/electrode interface. Both electrodes were measured in the redox solution of [Fe(CN)_6_]^3−/4−^ (5 mM) containing 1 M KNO_3_, using the frequency range of 100 kHz–0.1 Hz, to evaluate the charge transfer resistance (Rct) of electrodes which corresponds to the Randles equivalent circuit (Figure 7 inset). Rs represents solution resistance, Rct is charge transfer resistance, Cdl is double-layer capacitance, and W is Warburg impedance. The Nyquist plots (Figure 7) demonstrate the semicircles of CPE and MCPE.

The Rct values for CPE and MCPE were 15.32 kΩ and 6.84 kΩ, respectively. The Rct value for CPE that is the largest, indicates a very slow electron transfer rate between the redox solution and the electrode interface. The Rct value offered at MCPE implies fast charge transfer. The results suggest that the nanoparticles’ presence can facilitate electron transfer between the electrode surface and the redox solution, thereby increasing electro-conductibility. Hence, Fe_3_O_4_ nanoparticle was very efficient for developing an electrochemical sensor for the analysis of phenolic compounds [23,42,43].

### 3.6. Application of Gold Screen-Printed Electrode for Rapid Validation Test of Phenolic Compounds Using Cyclic Voltammetry

**Figure 8:**
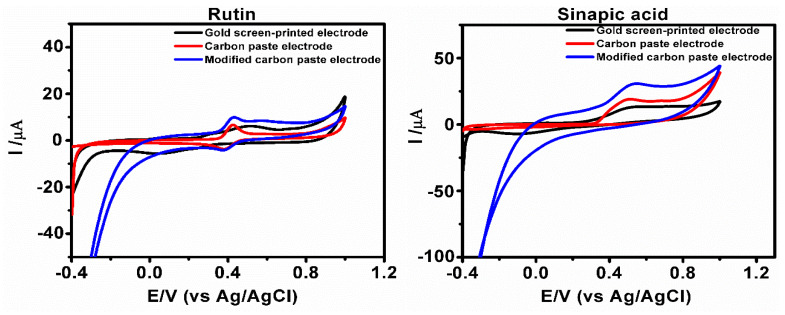
Cyclic voltammograms show an overlay of the gold screen-printed electrode with CPE and MCPE of the phenolic compounds rutin and sinapic acids in 0.5 M ABS of pH 4.8 at a scan rate of 0.2 V/s with a reversible scanning potential range of −0.4 to 1.0 V.

The gold screen-printed electrode was applied in the detection of rutin and sinapic acid using cyclic voltammetry technique in 0.5 mol L^−1^ ABS with a pH value of 4.8, at a scan rate of 0.2 V/s in a reversible potential sweep range of −0.4 to +1.0 V. This analysis was performed as a rapid test for the detection of these phenolic compounds and to compare the results with the CPE and MCPE because of its reproducibility, sensitivity, accuracy, and avoidance of preparation and cleaning process. The voltammograms of the rutin and sinapic acid on the electrode surface (Figure 8) indicate an overlay of the gold screen-printed electrode, CPE and MCPE. The sensitivity of the gold screen-printed electrode to the concentration of the phenolic compounds is compared to CPE and MCPE used, using the current density (J) = Current Intensity (A)/Cross-sectional Area (cm^2^). The surface area was taken using π r^2^ and divided by the value of current response for sinapic and rutin from the three electrodes used. For sinapic acid, the current density obtained is 0.1012 × 10^−3^ A/cm^2^ for gold screen-printed electrode, 0.1541 × 10^−3^ A/cm^2^ for CPE and 0.2466 × 10^−3^ A/cm^2^ for MCPE, for rutin the current density obtained is 0.0499 × 10^−3^ A/cm^2^ for gold screen-printed electrode, 0.0538 × 10^−3^ A/cm^2^ for CPE, and 0.0801 × 10^−3^ A/cm^2^ for MCPE. The result could also be suggested that the gold screen-printed active mass surface area is smaller than that of the CPE and MCPE. The use of the gold screen-printed electrode is important for future applications in the manufacture of electrochemical food sensor devices, which can detect different compounds present in food substances.

### 3.7. Effect of Concentration on the Phenolic Compounds Oxidation at CPE and MCPE

The effect of increasing the concentration of the phenolic compounds on their oxidation signals at MCPE was studied using DPV. The results were used to determine the limit of detection (LOD) and limit of quantification (LOQ) of the voltammetric method optimized for the quantification of the phenolic compounds on the modified carbon paste electrode surface. The phenolic compounds used in this work were investigated in a range of concentration from 0.3 × 10^−6^–13.0 × 10^−6^ M. The Equations (1) and (2) were used to calculate the limit of detection and limit of quantification of the phenolic compounds using the peak currents, respectively.

LOD = 3 * Sa/b,(2)

LOQ = 10Sa/b(3)

where “b” is the slope of our calibration curve, and “Sa” represents the standard deviation.

The recorded oxidation signals of the phenolic compounds increased with a gradual increase in the concentration of the phenolic compounds ranging from 0.3 × 10^−6^–13.0 × 10^−6^ M. The results obtained showed a linear relationship between peak currents and the change in concentration of the phenolic compounds. The following are the linear regression equations of the phenolic compounds: Ip = 1.3982 C + 1.2362 (Ip: µA, C: mol L^−1^ and R^2^ = 0.9865) for sinapic acid, Ip = 0.1457 C + 0.7410 (Ip: µA, C: mol L^−1^ and R^2^ = 0.9851) for syringic acid, and Ip = 0.7163 C + 0.6859 (Ip: µA, C: mol L^−1^ and R^2^ = 0.9860) for rutin. The proposed method for the phenolic compounds detection limit is compared with the maximum levels of antioxidants, within a range of 20 to 1000 ppm (20 to 1000 mg L^−1^) that are permitted within the guidelines for food taken within the EU and North America [44]. The detection limits of the developed DPV method for the phenolic compounds were calculated. The values were compared with other data reported by other research groups (Table 4), where rutin was reported with other acids, but less or no work has been reported on sinapic acid and syringic acid, respectively.

### 3.8. Reproducibility, Repeatability, and Stability

The sensor’s reproducibility was investigated by using the MCPE for the determination of 0.9 × 10^−3^ M for sinapic acid, 1.0 × 10^−3^ M for syringic acid, and 0.3 × 10^−3^ M for rutin, respectively, using DPV in 0.5 M ABS pH 4.8. Seven independent electrodes were used to determine each analyte. The relative standard deviations (RSD) were found to be 4.2% for sinapic acid, 3.6% for syringic acid, and 4.6% for rutin (Figure S7), hence showing good reproducibility. The repeatability was also investigated using seven prepared modified electrodes in seven prepared samples for each analyte, and the relative standard deviations of the peak currents were found to be 3.1% for sinapic acid, 4.2% for syringic acid, and 4.2% for rutin, hence indicating good repeatability (Figure S7). Three modified electrodes were prepared for the determination of the stability of the sensor. A potential of 0.6 V was applied using the chronoamperometry method for each analyte with the above concentration at the modified electrode for 30 min, respectively (Figure S8). These potentials are comparable to the phenolic compounds’ oxidation potentials of the CV analysis results done previously from this study. The amperometric response observed remained constant throughout the experiment. The surface of the electrodes did not undergo any fouling; hence, this attests to the proposed sensor’s stability [43]. The stability was again analyzed using the above concentrations of the analytes using DPV on the first day using two modified electrodes, which was then stored for 10 days at room temperature in the laboratory. The electrodes were then used to determine the same concentration of the phenolic compounds after 10 days. The respective voltammograms on the 1st day and 10th day of the modified electrodes (Figure S8) demonstrated good stability with relative standard deviation values of 3.85% for sinapic acid, 4.54% for syringic acid, and 7.17% for rutin, respectively.

### 3.9. Selectivity of the Electrode

To evaluate the selectivity of the fabricated sensor in identifying the analytes of interest, the effects of possible interferences were investigated by analyzing a standard solution of 0.9 × 10^−3^ M for sinapic, 1.0 × 10^−3^ M for syringic, and 0.3 × 10^−3^ M for rutin respectively in 0.5 M ABS pH 4.8. Common inorganic ions such as K^+^, Cl^−^, Fe^+3^, and Ca^+2^, had no significant interference in determining the phenolic compounds with an RSD of the oxidation peaks obtained to be less than 5% (Table 5). Other potential electroactive organic interferences, such as caffeic acid and 4-hydroxybenzoic acid, which may co-exist with the analytes, were also examined. These organic interferences with their concentration increased about 500-fold excess did not meaningfully change the oxidation peak currents of the analytes of interest, and the RSD values obtained were less than 5% (Table 5). Therefore, MCPE can be used for the selective determination of sinapic acid, syringic acid, and rutin.

### 3.10. Simultaneous Detection of the Phenolic Compounds at MCPE

**Figure 9:**
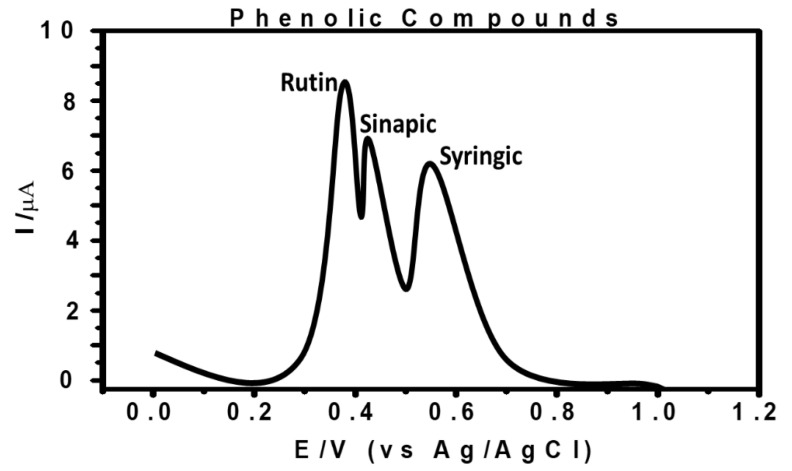
Differential pulse voltammogram showing three phenolic compounds’ simultaneous determination with 4.5 × 10^−3^ M for sinapic acid, 5.1 × 10^−3^ M for syringic acid, and 1.6 × 10^−3^ M for rutin respectively at MCPE in 0.5 M ABS with pH 4.8, recorded at 0 V to +1.0 V.

DPV was used to study all the three phenolic compounds (sinapic acid, syringic acid, and rutin) simultaneously to observe their electro-oxidation behavior at MCPE (Figure 9). The analytes presented oxidation potentials similar and within the same potential with the oxidation potentials of other results in this study, where the analytes were analyzed individually. This result showed that the modified electrode has an ability to detect the presence of all the three phenolic acids simultaneously in the solution.

### 3.11. Application of CPE and MCPE on Phenolic Compounds Detection in Red and White Wines

The determination of the presence of the phenolic compounds in red and white samples, respectively, was done using MCPE. This study was carried out with diluted 10 mL samples of red and white wine, which served as blanks. CV and DPV of the red and white wine samples were analyzed (Figure S9) using the modified electrode without the presence of the standard phenolic compounds to observe if these particular commercial wine samples that were purchased contained sinapic, syringic, and rutin. The sensor used detected that the wine samples did not contain the presence of the phenolic compounds of interest; instead, they contained other antioxidants or sulphites, indicating oxidation potentials that seem to appear as the analyte of interest. The CV of the wine samples showed little anodic peaks, while the DPV was able to indicate the presence of some other compounds, which were not the standard phenolic compounds used except sinapic acid in red wine, which has a very low concentration. Aliquots of the known concentration of phenolic compounds (sinapic, syringic, and rutin) through the standard addition method were then added to the wine samples to observe the modified electrode’s detection ability. The wine samples analysis was carried out with DPV (Table 6), and the results of the analysis of wine samples suggest the activity of oxidation that occurred. However, as the spiked wine concentrations were increased, there was an increase in the oxidation peaks of the phenolic compounds in the red and white wine. The modified electrode detected the presence of the added standard phenolic compounds in both white and red wine samples with recoveries at almost 100%.

## 4. Conclusions

The fabrication of this electrochemical sensor for detecting the selected phenolic compounds (sinapic acid, syringic acid, and rutin) using Fe_3_O_4_ nanoparticles to modify CPE is first reported in this study after a careful check of other reported articles. The results obtained by CV, DPV, and EIS showed that the CPE modified with Fe_3_O_4_ nanoparticles increased the peak current, leading to increased sensitivity to bare CPE. EIS analysis confirmed that MCPE exhibited increased electro-conductibility, thus enhancing the electron transfer between the electrode surface and the redox solution. The MCPE showed high sensitivity, selectivity, reproducibility, repeatability, and stability towards the determination of the phenolic compounds. The CPE and MCPE were used to compare commercial gold screen-printed electrodes for rapid detection of the phenolic compounds, confirmed by their current density that the MCPE had higher current density than CPE and gold screen-printed electrodes. The fabrication of this electrochemical sensor was simple and cost- and time-effective. The LOD and LOQ results were compared to other literature’ sensors; syringic acid is first reported in this work. The electrochemical sensor was applied for real sample analysis to determine phenolic compounds in red and white wine samples. The results found are within the maximum concentrations of 20 to 1000 ppm (20 to 1000 mg L^−1^) antioxidant levels permitted for phenolic compounds in food samples within the EU and North America.

## Figures and Tables

**Table 1:** Linear regression equation of anodic peak potentials E_pa_ and pH of the phenolic compounds with their slopes respectively reported for cyclic voltammetry method employed in determining the pH on the electrochemical behavior of the phenolic compounds at bare CPE and MCPE.

Phenolic Compounds	Regression Equation of Anodic Peak Potentials E_pa_ and the pH	R^2^ Value	Slope of E_pa_/pH mV/pH	Nernstian Value mV/pH
Sinapic acid (CPE)	E_pa_ (V) = 0.7138–0.0398 pH	0.9924	40	59
Sinapic acid (MCPE)	E_pa_ (V) = 0.6953–0.0338 pH	0.996	34	59
Syringic acid (CPE)	E_pa_ (V) = 0.8883–0.0424 pH	0.9590	42	59
Syringic acid (MCPE)	E_pa_ (V) = 1.2148–0.0366 pH	0.9700	37	59
Rutin (CPE)	E_pa_ (V) = 0.6621–0.0486 pH	0.9939	49	59
Rutin (MCPE)	E_pa_ (V) = 0.5787–0.0255 pH	0.9833	26	59

**Table 2:** Linear regression equations show the dependence of redox peak current Ip on the square root of scan rate ν^1/2^ (V/s)^1/2^ for controlled diffusion and dependence of redox peak current Ip on scan rate ν (V/s) for controlled adsorption for phenolic compounds at bare CPE and MCPE with their slopes and R-square values.

Phenolic Compounds	Regression Equation I_pa_ on ν^1/2^ (V/s)^1/2^ Controlled Diffusion	R^2^ Value	Regression Equation I_pa_ versus Scan Rate ν (V/s) Controlled Adsorption	R^2^ Value
Sinapic acid (CPE)	I_pa_ (μA) = 7.7340 ν^1/2^ − 1.5106	0.9545	I_pa_ (µA) = 9.4885 ν − 0.1137	0.9988
Sinapic acid MCPE)	I_pa_ (μA) = 17.1927 ν^1/2^ − 0.4542	0.9848	I_pa_ (µA) = 20.6984 ν + 2.7275	0.9880
Syringic acid (CPE)	I_pa_ (μA) = 1.8253 ν^1/2^ + 0.5594	0.9923	I_pa_ (µA) = 2.1349 ν + 0.9093	0.9333
Syringic acid (MCPE)	I_pa_ (μA) = 3.0278 ν^1/2^ + 0.3438	0.9977	I_pa_ (µA) = 3.5624 ν + 0.9202	0.9510
Rutin (CPE)	I_pa_ (μA) = 1.3556 ν^1/2^ − 0.1777	0.9805	I_pa_ (µA) = 1.6414 ν + 0.4267	0.9964
Rutin (MCPE)	I_pa_ (μA) = 5.5898 ν^1/2^ − 0.5157	0.9906	I_pa_ (µA) = 6.7137 ν + 0.5218	0.9885

CPE = Carbon Paste Electrode, MCPE = Iron oxide modified carbon paste electrode, I_pa_ = Anodic peak current, ν^½^ = Square root of scan rate, (V/s)^1/2^ = square root of volt per second.

**Table 3:** Linear regression equation showing the logarithm of anodic peak current and logarithm of scan rate (log Ip versus log ν (V/s) for the phenolic compounds at CPE and MCPE with their slopes and R-square values.

Phenolic Compounds	Regression Equation log I_pa_ Versus log ν (V/s)	R^2^ Value
Sinapic acid (CPE)	Log I_pa_ (µA) = 0.6192 Log ν + 0.3387	0.9935
Sinapic acid (MCPE)	Log I_pa_ (µA) = 0.5090 Log ν + 1.2126	0.9888
Syringic acid (CPE)	Log I_pa_ (µA) = 0.49611 Log ν + 0.6904	0.9990
Syringic acid (MCPE)	Log I_pa_ (µA) = 0.5119 Log ν + 0.7833	0.9980
Rutin (CPE)	Log I_pa_ (µA) = 0.5168 Log ν + 0.4350	0.9976
Rutin (MCPE)	Log I_pa_ (µA) = 0.6581 Log ν + 0.7490	0.9987

**Table 4:** Limits of detection (LOD) and limit of quantification (LOQ) reported for the differential pulse voltammetry method employed in detecting phenolic compounds compared to other methods used.

Electrode	Method	Phenolic Compounds	Linear Dynamic Range(M)	Limit of Detection (M)	Limit of Quantification (M)	Ref.
Ni-GO/GCE	SQWV	Rutin	1.1 × 10^−8^ to 1.0 × 10^−6^	3.2 × 10^−9^	--	[13]
CTAC/Gr/PdNPs	SQWV	Rutin	0.02 × 10^−6^ to 1.0 × 10^−6^	0.005 × 10^−6^	--	[11]
GCE/EAuNPs/rGO/Naf	CV, LSV, EIS	Sinapic	20 × 10^−6^ to 200 × 10^−6^	33.43 × 10^−9^	--	[45]
CPE/Fe_3_O_4_ NPs	DPV	Rutin	0.3 × 10^−6^ to 3.0 × 10^−6^	0.8 × 10^−7^	2.5 × 10^−7^	This work
CPE/Fe_3_O_4_ NPs	DPV	Sinapic	0.9 × 10^−6^ to 8.0 × 10^−6^	2.2 × 10^−7^	6.7 × 10^−7^	This work
CPE/Fe_3_O_4_ NPs	DPV	Syringic	1.0 × 10^−6^ to 9.1 × 10^−6^	2.6 × 10^−7^	8.0 × 10^−7^	This work

CPE = Carbon Paste Electrode, MCPE = Iron oxide nanoparticles modified carbon paste electrode, mol L^−1^ = moles per liter, GCE/EAuNPs/rGO/Naf = Glassy Carbon Electrode, Electrochemically tuned gold nanoparticles and reduced graphene oxide (rGO) Ref. = References, Ni-GO/GCE = Nickel nanoparticles incorporated with graphene oxide composite-glassy carbon electrode, CTAC = cetyltrimethylammonium chloride, DPV = Differential Pulse Voltammetry, CV = Cyclic Voltammetry, SQWV = Square Wave Voltammetry.

**Table 5:** Effect of various interferences on the determination of sinapic, syringic, and rutin.

Interfering Species	Sinapic Acid (RSD%)	Syringic Acid (RSD%)	Rutin (RSD%)
K^+^	±4.99	±3.75	±4.03
Cl^−^	±1.95	±3.00	±4.78
Fe^+3^	±4.27	±4.76	±3.99
Ca^+2^	±4.34	±4.26	±2.20
caffeic acid	±4.85	±3.43	±4.98
4-hydroxybenzoic acid	±3.56	±3.40	±2.74

**Table 6:** Results of the determination of phenolic compounds in wine samples (red and white wine) using Fe_3_O_4_ nanoparticles modified CPE.

**Samples** **Red Wine**	**Rutin** Ip = 19.1695x + 0.0045, R^2^ = 0.9992				**Sinapic Acid** Ip = 10.6158x + 0.1531, R^2^ = 0.9995			
	**Added** **(mmol L^−1^)**	**Found** **(mmol L^−1^)**	**Recovery** **(%)**	**Relative** **Error**	**Added** **(mmol L^−1^)**	**Found** **(mmol L^−1^)**	**Recovery** **(%)**	**Relative** **Error**
	0	Undetected	-	-	0	0.0002	-	-
	0.03	0.0294	98	±2	0.03	0.0295	98.33	±1.67
	0.05	0.0504	100.8	±0.8	0.05	0.0503	100.6	±0.6
**Syringic acid** Ip = 9.5979x + 0.198, R^2^ = 1								
	**Added** **(mmol L^−1^)**	**Found** **(mmol L^−1^)**	**Recovery** **(%)**	**Relative** **Error**				
	0	0	-	-				
	0.03	0.03	100	±0				
	0.05	0.05	100	±0				
**Samples** **White Wine**	**Rutin** Ip = 26.8763x + 0.0022, R^2^ = 0.9999				**Sinapic acid** Ip = 19.2895x + 0.00995, R^2^ = 0.9960			
	**Added** **(mmol L^−1^)**	**Found** **(mmol L^−1^)**	**Recovery** **(%)**	**Relative Error**	**Added** **(mmol L^−1^)**	**Found** **(mmol L^−1^)**	**Recovery** **(%)**	**Relative** **Error**
	0	Undetected	-	-	0	Undetected	-	-
	0.03	0.0302	100.67	±0.67	0.03	0.0313	104.3	±4.33
	0.05	0.0499	99.8	±0.2	0.05	0.0492	98.4	±1.6
**Syringic acid** Ip = 19.7829x + 0.0047, R^2^ = 0.9992								
	**Added** **(mmol L^−1^)**	**Found** **(mmol L^−1^)**	**Recovery** **(%)**	**Relative** **Error**				
	0	Undetected	-	-				
	0.03	0.0306	102	±2				
	0.05	0.0496	99.2	±0.8				

## Supplementary Materials

The following are available online at https://www.mdpi.com/2072-666X/12/3/312/s1. Figure S1: The chemical reaction process showing the oxidation of phenolic compounds having one peak in the reaction path (A) and the oxidation of phenolic compounds having two peaks in the reaction path (B). Figure S2: Cyclic voltammograms at (A) CPE and (B) MCPE, respectively, in 1 mM K_4_[Fe(CN)_6_] of 0.1 M KCl by varying scan rates (0.1–0.2 V/s). The corresponding Plots of I_pa_ vs. *v*^1/2^ at (C) CPE and (D) MCPE. Figure S3: The linear regression plots for both CPE and MCPE to show the effect of pH on the electrochemical behavior of the phenolic compounds, in 0.5 M acetate buffer of pH 2.6, 3.8, 4.8, 5.6, 6.5, 7.4, 8.4, and 9.2, at a scan rate of 0.2 V/s. Figure S4: Linear regression plots of phenolic compounds showing the dependence of redox (anodic and cathodic) peak current Ip on the square root of scan rate ν^1/2^ (V/s)^1/2^. The plots represent controlled diffusion at CPE and MCPE in 0.5 M of ABS with pH 4.8, the scan rate of 0.03, 0.06, 0.09, 0.12, 0.15, 0.18, 0.20, 0.25, 0.3, 0.35, and 0.40 V/s. Figure S5: Linear regression plots showing the dependence of redox (anodic and cathodic) peak current on scan rate Ip versus scan rate ν (V/s) for controlled adsorption. The plots represent controlled adsorption at CPE and MCPE in 0.5 M of ABS with pH 4.8 and the scan rate of 0.03, 0.06, 0.09, 0.12, 0.15, 0.18, 0.20, 0.25, 0.3, 0.35, and 0.40 V/s, respectively, with a reversible scanning potential range of −0.4 to 1.0 V. Figure S6: The linear relationship plots of the logarithm of peak current and logarithm of scan rate (log I_pa_ versus log ν) for phenolic compounds. The plots represent peak current and logarithm of scan rate at CPE and MCPE. Figure S7: Differential voltammograms showing the reproducibility and repeatability of MCPE for the determination of the phenolic compounds, respectively. Figure S8: A steady amperometric current response for determination of stability of the sensor for phenolic compounds detection in (0.5 M ABS of pH 4.8), for 30 min, at MCPE using 0.6 V potential. Differential pulse voltammograms show the determination of 0.9 × 10^−3^ M for sinapic, 1.0 × 10^−3^ M for syringic, and 0.3 × 10^−3^ M for rutin, respectively, in 0.5 M ABS pH 4.8 for 1st day and 10th day to show the stability of the modified electrode. Figure S9: Cyclic voltammograms and Differential pulse voltammograms showing the determination of red and white wine real samples only using the modified electrode in 0.5 M ABS pH 4.8 as supporting electrolyte.
