# Supplementary material for: Electrochemical Sensor for Determination of Various Phenolic Compounds in Wine Samples Using Fe3O4 Nanoparticles Modified Carbon Paste Electrode
Source: Micromachines (Basel). 2021 Mar 17;12(3):312. doi: 10.3390/mi12030312 (PMC8002544; doi:10.3390/mi12030312)
Supplement: Supplementary file 1 [file micromachines-12-00312-s001.pdf]

micromachines

MDPI

Article

Electrochemical Sensor for Determination of Various Phenolic Compounds in Wine samples using Fe_3O_4 nanoparticles modified Carbon Paste Electrode

Pwadubashiyi C. Pwavodi [1,*], Vasfiye H. Ozyurt [2], Suleyman Asir [3] and Mehmet Ozsoz [1]

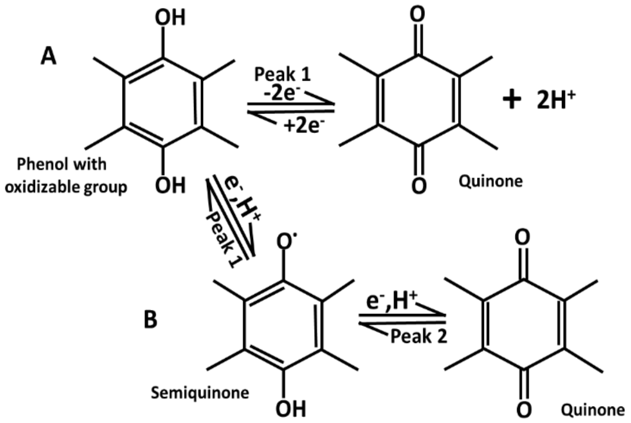

Figure S1. The chemical reaction process showing the oxidation of phenolic compounds having one peak in the reaction path (A) and the oxidation of phenolic compounds having two peaks in the reaction path (B).

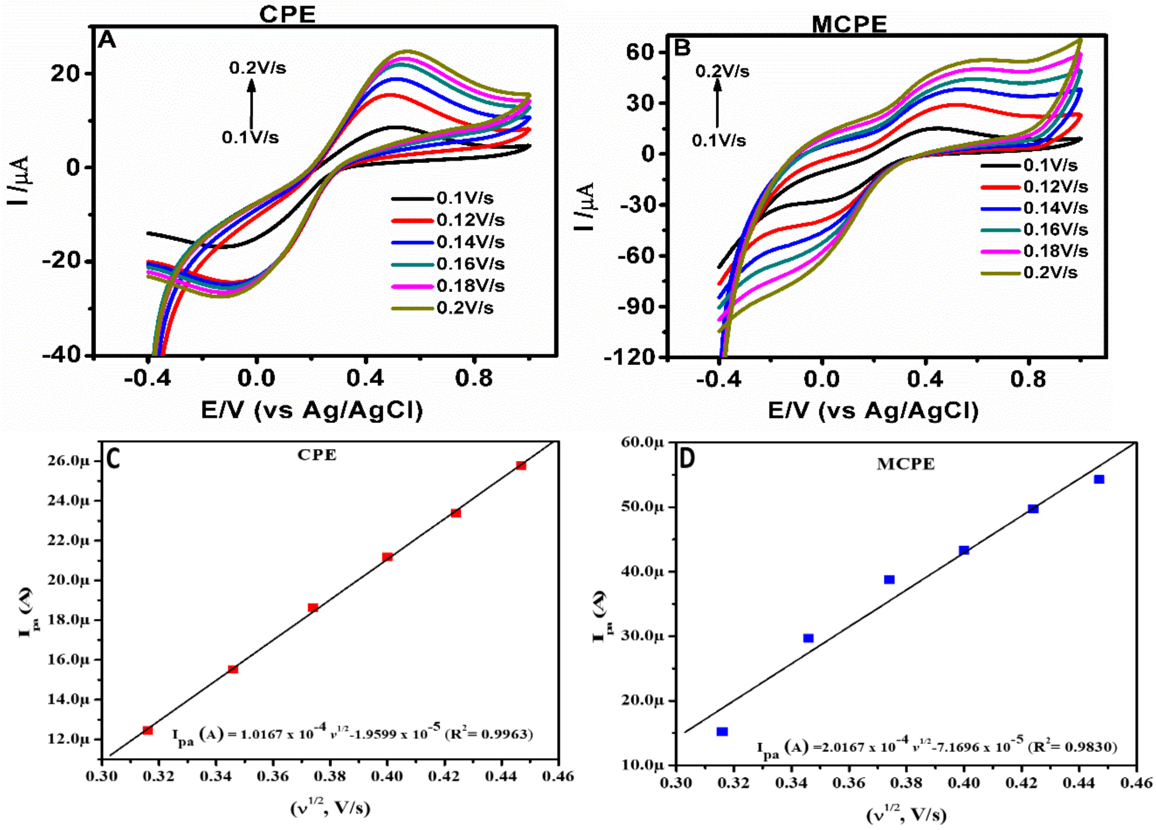

Figure S2. Cyclic voltammograms at (A) CPE and (B) MCPE, respectively in 1 mM $K_4[Fe(CN)_6]$ of 0.1 M KCl by varying scan rates (0.1–0.2 V/s). The corresponding Plots of I_{pa} vs $v^{1/2}$ at (C) CPE and (D) MCPE.

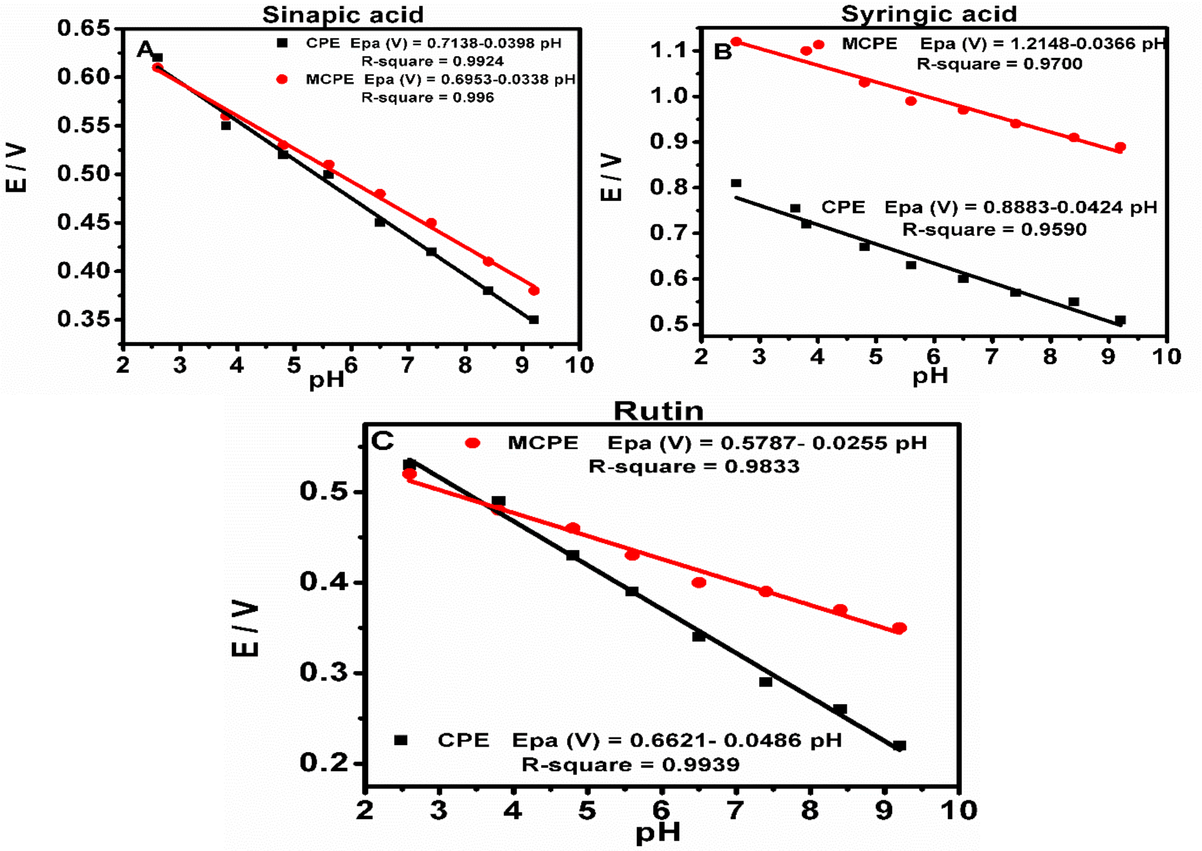

Figure S3. The linear regression plots for both CPE and MCPE to show the effect of pH on the electrochemical behavior of the phenolic compounds, in 0.5 M acetate buffer of pH 2.6, 3.8, 4.8, 5.6, 6.5, 7.4, 8.4 and 9.2, at a scan rate of 0.2 V/s.

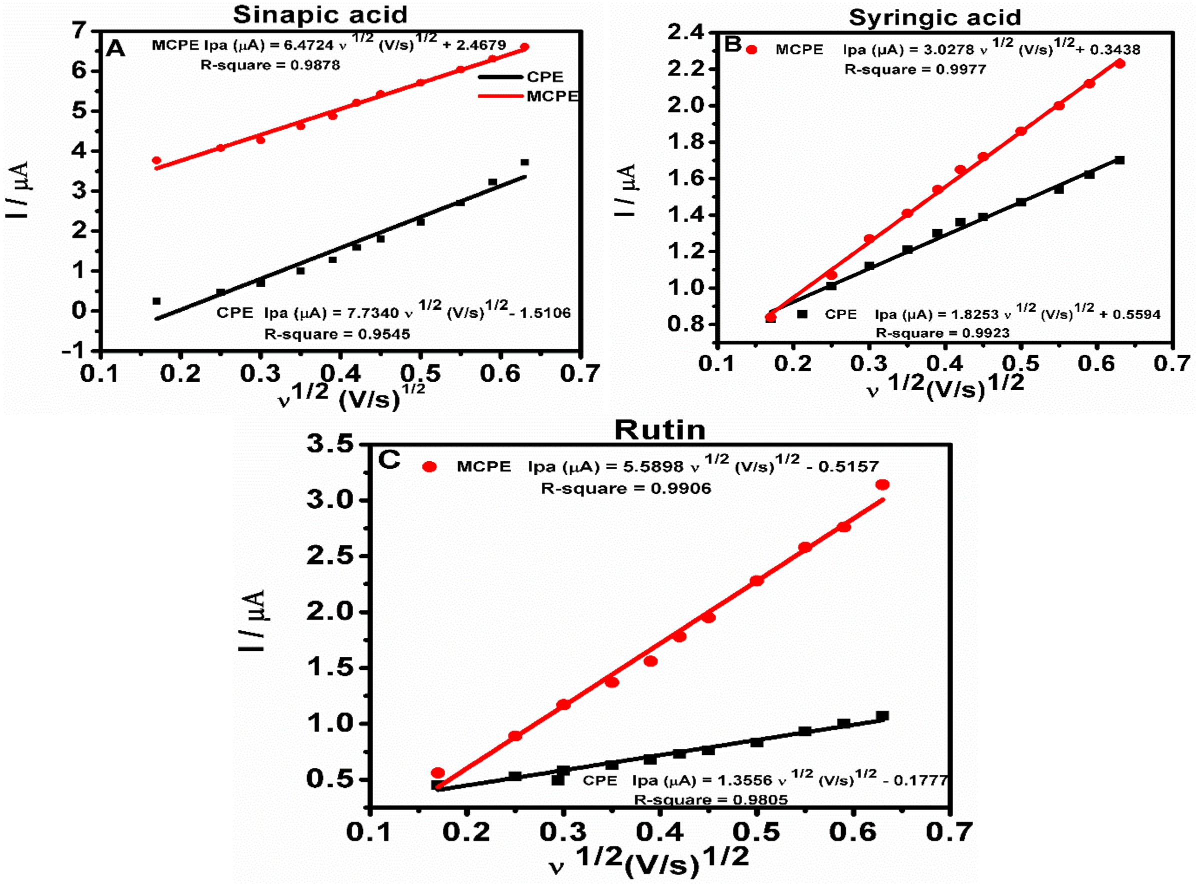

Figure S4. Linear regression plots of phenolic compounds showing the dependence of redox (anodic and cathodic) peak current Ip on the square root of scan rate $\nu^{1/2}$(V/s)$^{1/2}$. The plots, represents controlled diffusion at CPE and MCPE in 0.5 M of ABS with pH 4.8, scan rate of 0.03, 0.06, 0.09, 0.12, 0.15, 0.18, 0.20, 0.25, 0.3, 0.35 and 0.40 V/s.

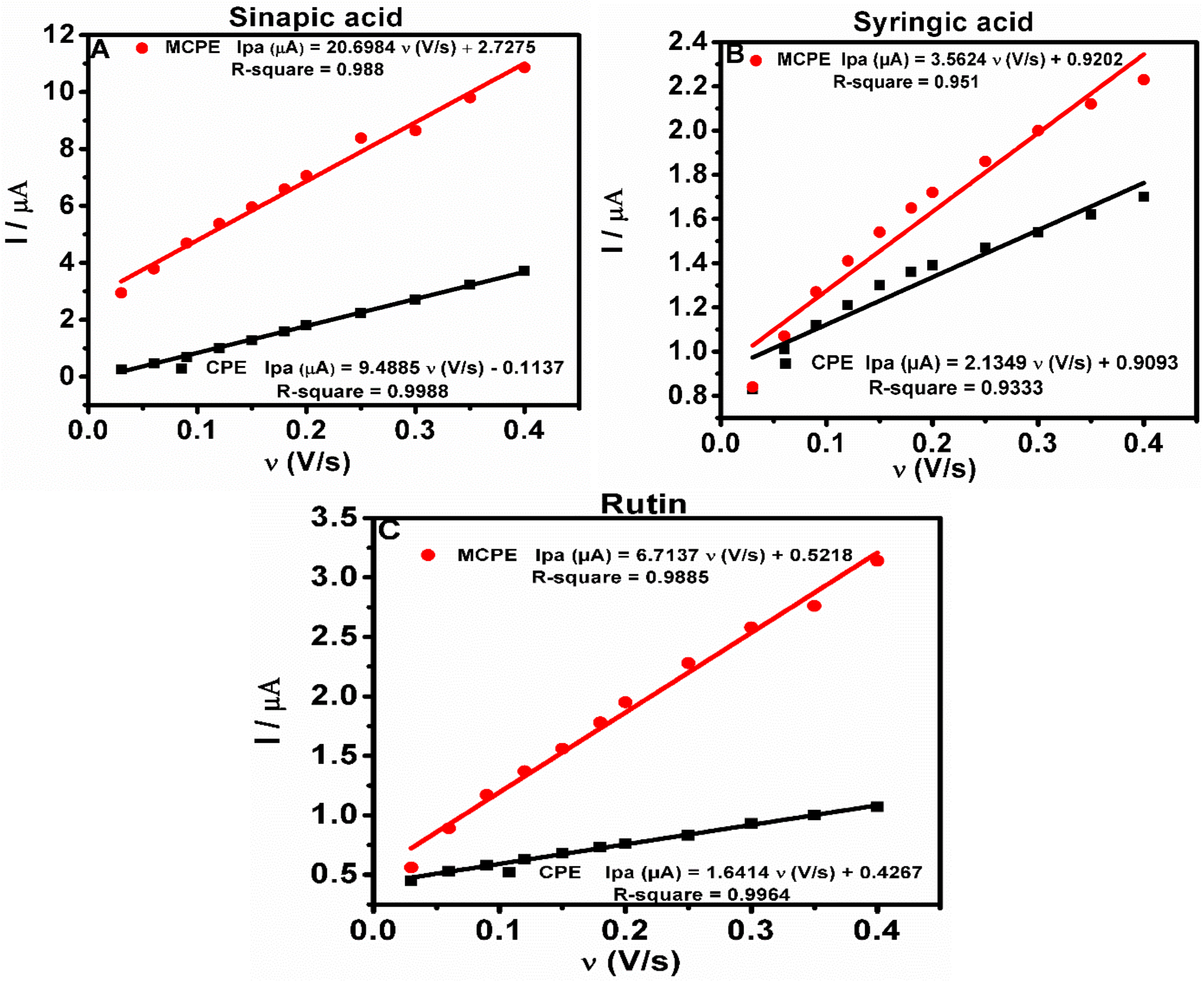

Figure S5. Linear regression plots showing the dependence of redox (anodic and cathodic) peak current on scan rate Ip versus scan rate ν (V/s) for controlled adsorption The plots, represents controlled adsorption at CPE and MCPE in 0.5 M of ABS with pH 4.8, scan rate of 0.03, 0.06, 0.09, 0.12, 0.15, 0.18, 0.20, 0.25, 0.3, 0.35 and 0.40 V/s respectively with a reversible scanning potential range of -0.4 to 1.0 V.

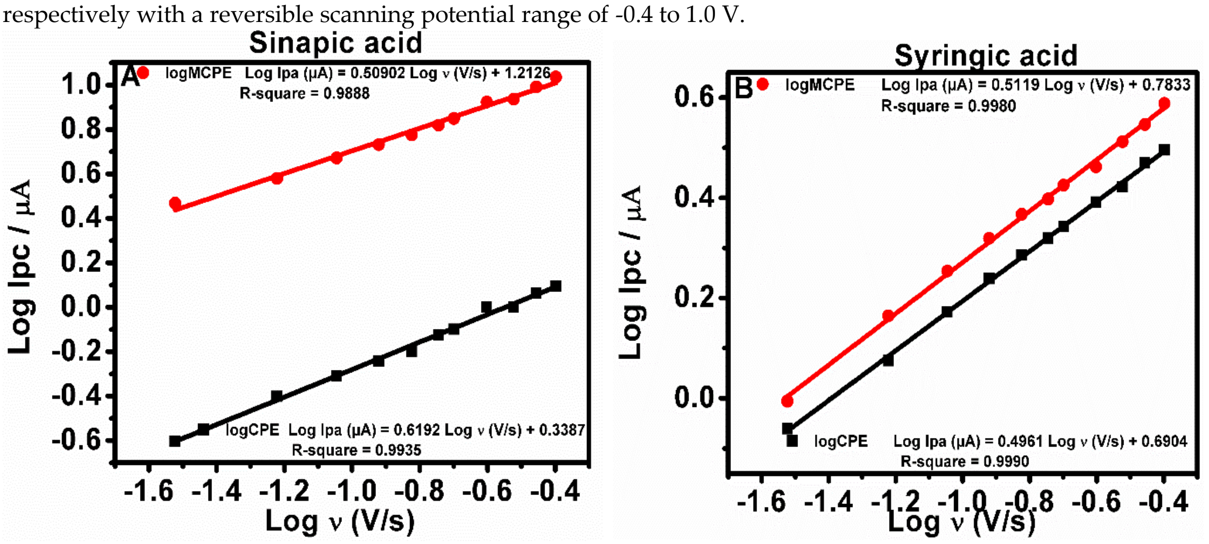

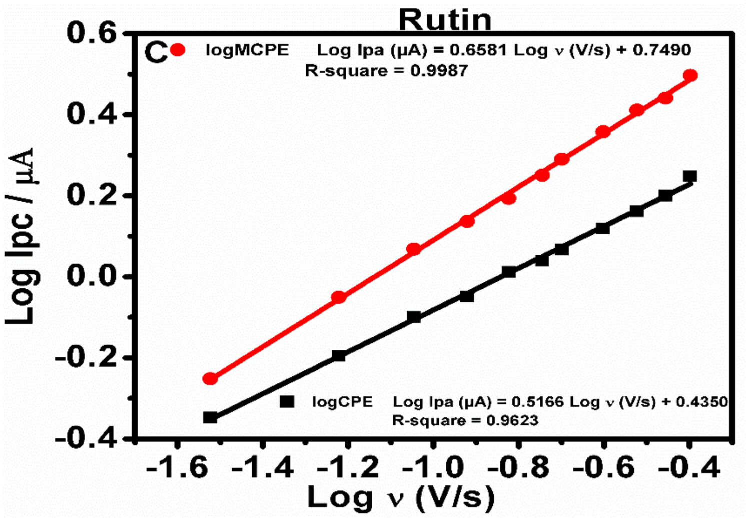

Figure S6. The linear relationship plots of logarithm of peak current and logarithm of scan rate (log I_{pa} versus log ν) for phenolic compounds. The plots, represents peak current and logarithm of scan rate at CPE and MCPE.

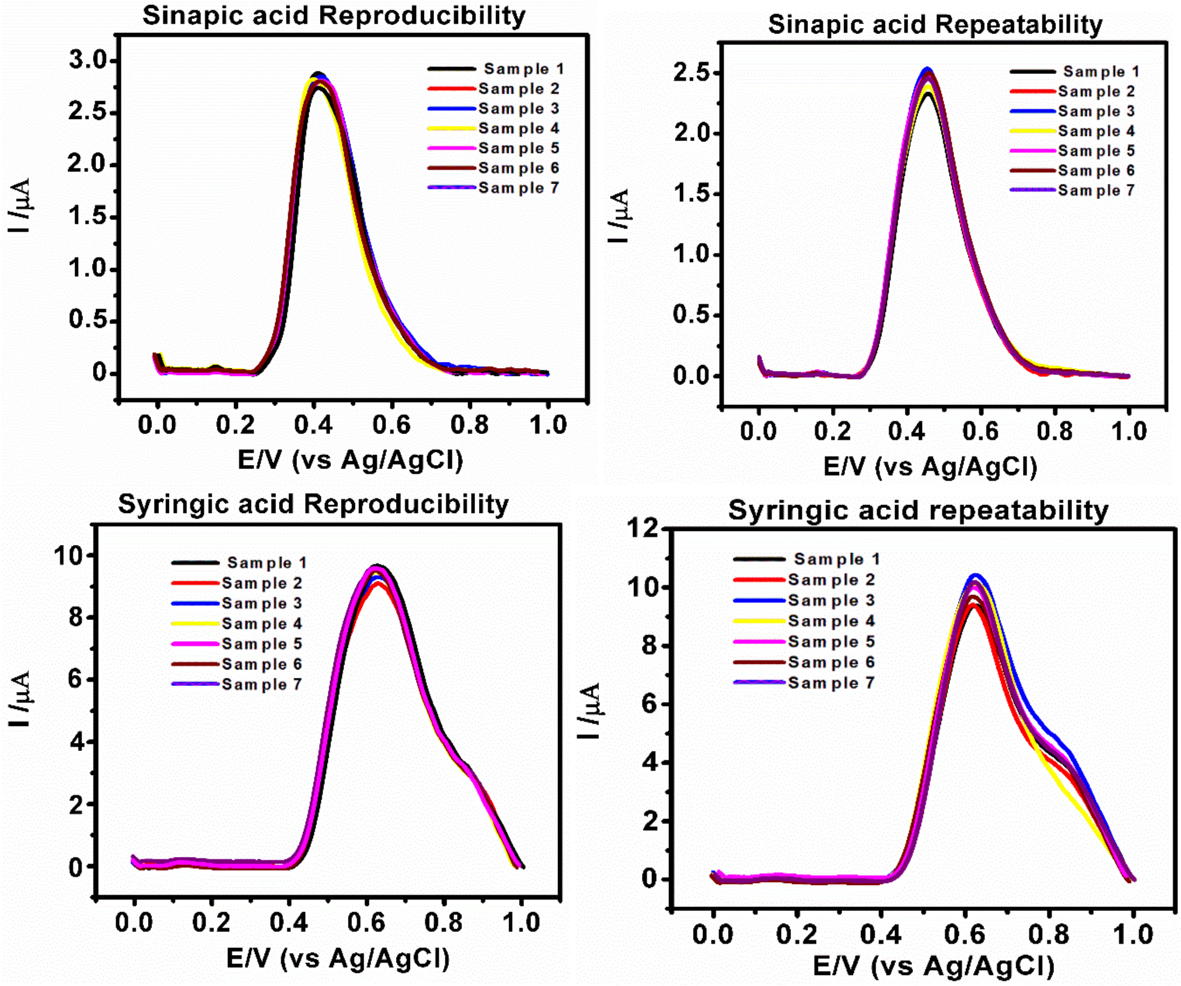

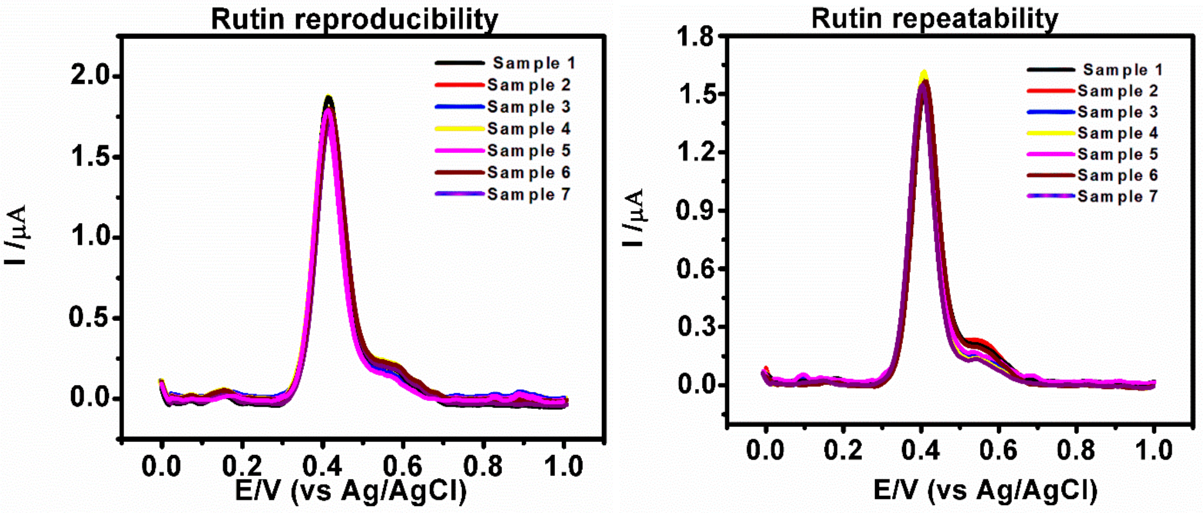

Figure S7. Differential voltammograms showing the reproducibility and repeatability of MCPE for the determination of the phenolic compounds respectively.

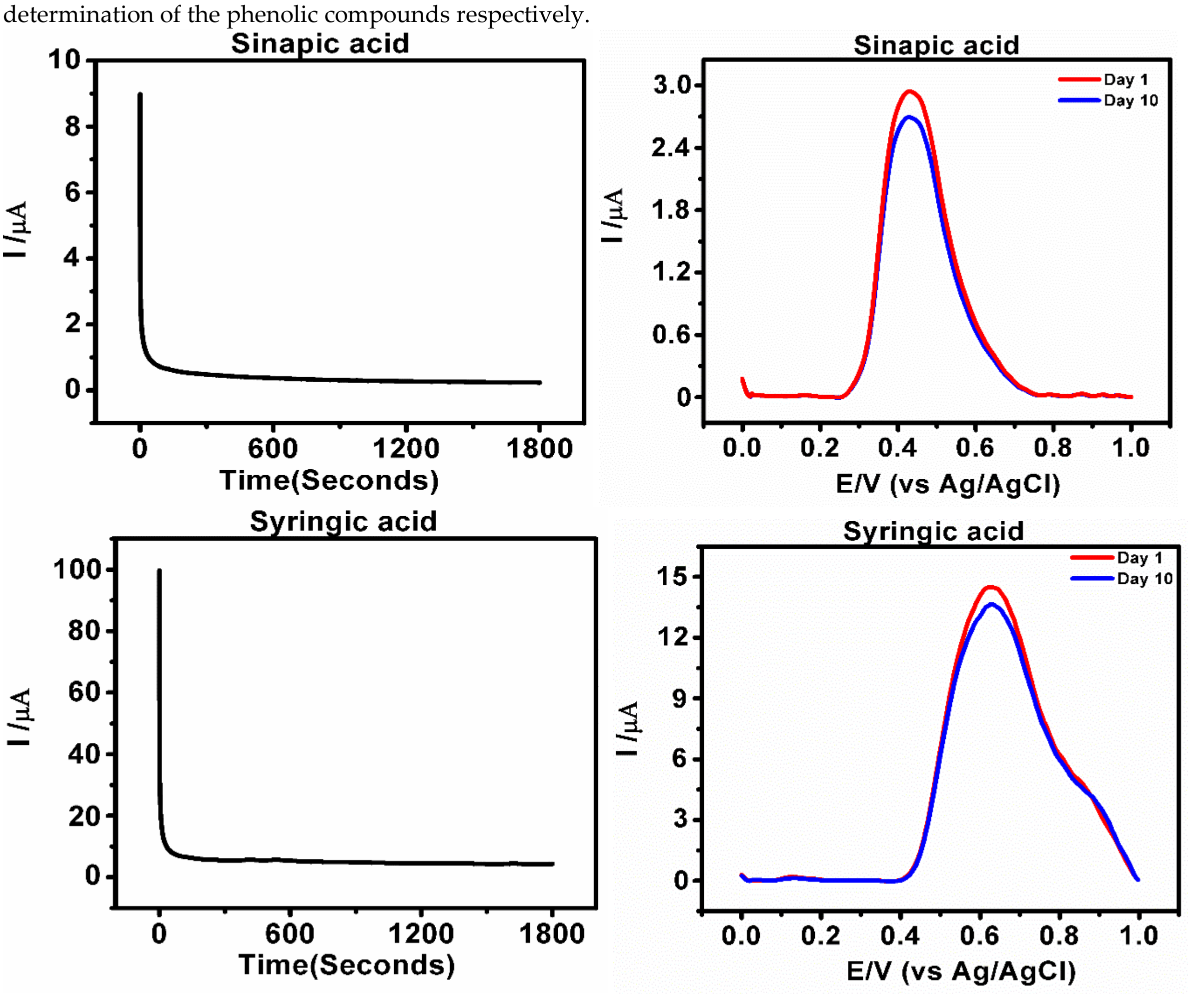

micromachines

MDPI

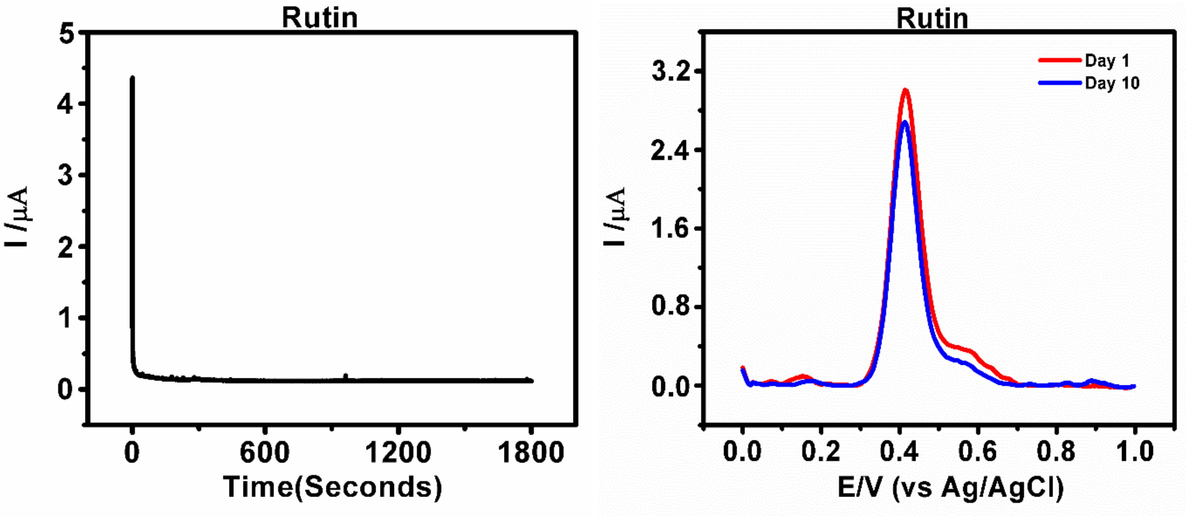

Figure S8. A steady amperometric current response for determination of stability of the sensor for phenolic compounds detection in (0.5 M ABS of pH 4.8), for 30 minutes, at MCPE using 0.6V potential. Differential pulse voltammograms shows the determination of 0.9 × 10^{-3} M for sinapic, 1.0 × 10^{-3} M for syringic and 0.3 × 10^{-3} M for rutin respectively in 0.5M ABS pH 4.8 for 1st day and 10th to show stability of the modified electrode.

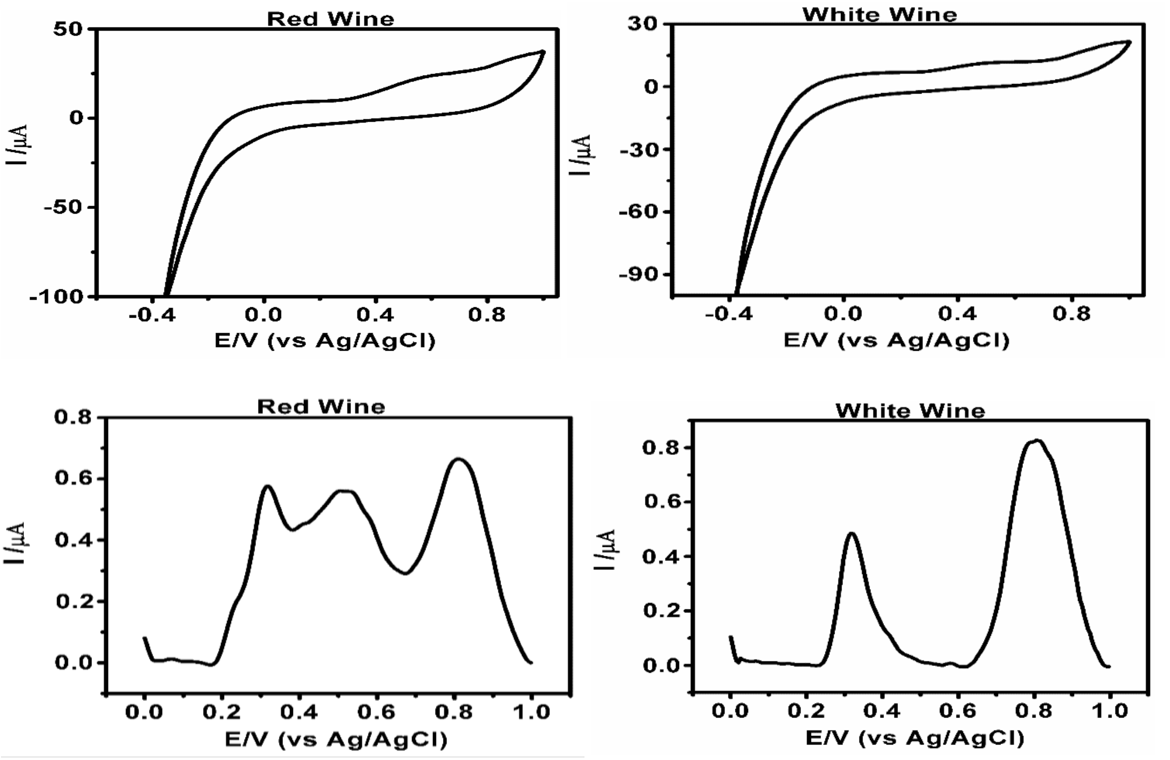

Figure S9. Cyclic voltammograms and Differential pulse voltammograms showing the determination of red and white wine real samples only using the modified electrode in 0.5M ABS pH 4.8 as supporting electrolyte.

Publisher's Note: MDPI stays neutral with regard to jurisdictional claims in published maps and institutional affiliations.

© 2020 by the authors. Submitted for possible open access publication under the terms and conditions of the Creative Commons Attribution (CC BY) license (http://creativecommons.org/licenses/by/4.0/).

micromachines
